# ARTS-DB 2.0: an expanded database for target-directed genome mining across bacteria and fungi

**DOI:** 10.1093/database/baag060

**Published:** 2026-09-25

**Authors:** Turgut Mesut Yılmaz, Semih Erdogmus, Caner Bağcı, Nadine Ziemert

**Affiliations:** Translational Genome Mining for Natural Products, Interfaculty Institute of Microbiology and Infection Medicine Tübingen (IMIT), Interfaculty Institute for Biomedical Informatics (IBMI), University of Tübingen, Auf der Morgenstelle 24, Tübingen, Baden-Württemberg 72076, Germany; German Center for Infection Research (DZIF), Partner Site Tübingen, Auf der Morgenstelle 24, 72076, Tübingen, Germany; Translational Genome Mining for Natural Products, Interfaculty Institute of Microbiology and Infection Medicine Tübingen (IMIT), Interfaculty Institute for Biomedical Informatics (IBMI), University of Tübingen, Auf der Morgenstelle 24, Tübingen, Baden-Württemberg 72076, Germany; German Center for Infection Research (DZIF), Partner Site Tübingen, Auf der Morgenstelle 24, 72076, Tübingen, Germany; Translational Genome Mining for Natural Products, Interfaculty Institute of Microbiology and Infection Medicine Tübingen (IMIT), Interfaculty Institute for Biomedical Informatics (IBMI), University of Tübingen, Auf der Morgenstelle 24, Tübingen, Baden-Württemberg 72076, Germany; German Center for Infection Research (DZIF), Partner Site Tübingen, Auf der Morgenstelle 24, 72076, Tübingen, Germany; Translational Genome Mining for Natural Products, Interfaculty Institute of Microbiology and Infection Medicine Tübingen (IMIT), Interfaculty Institute for Biomedical Informatics (IBMI), University of Tübingen, Auf der Morgenstelle 24, Tübingen, Baden-Württemberg 72076, Germany; German Center for Infection Research (DZIF), Partner Site Tübingen, Auf der Morgenstelle 24, 72076, Tübingen, Germany

## Abstract

The increasing prevalence of antimicrobial resistance requires efficient strategies to prioritize biosynthetic gene clusters (BGCs) that may encode bioactive compounds with new modes of action. Target-directed genome mining (TDGM) addresses this challenge by leveraging self-resistance determinants encoded within or adjacent to BGCs to generate target hypotheses and prioritize candidate clusters. ARTS and FunARTS automate TDGM for bacterial and fungal genomes by detecting resistance-associated evolutionary signals, including duplicated essential targets, atypical phylogenetic patterns, and BGC co-localization. The Antibiotic Resistant Target Seeker database (ARTS-DB) was originally developed to enable exploration of precomputed TDGM outputs for bacteria. Here, we present ARTS-DB 2.0, a major update that integrates precomputed ARTS and FunARTS results into a single cross-domain resource. ARTS-DB 2.0 contains TDGM outputs for 42 087 high-quality, non-redundant genomes (35 681 bacteria and 6406 fungi) and incorporates updated BGC annotations generated with antiSMASH 8.0 together with an expanded resistance model repertoire, including the NCBI AMR HMM catalog. The web interface supports flexible query construction and gene-centric exploration, and provides extensive cross-links to external protein, biosynthetic, and pharmacology resources (e.g. UniProt, MIBiG, ChEMBL, and DrugBank). ARTS-DB 2.0 enables rapid, large-scale investigation of resistance-associated biosynthetic patterns across bacteria and fungi, supporting resistance-aware target and BGC prioritization for natural product discovery.

**Database URL**: https://arts-db.cs.uni-tuebingen.de/

## Graphical Abstract

**Graphical Abstract:**
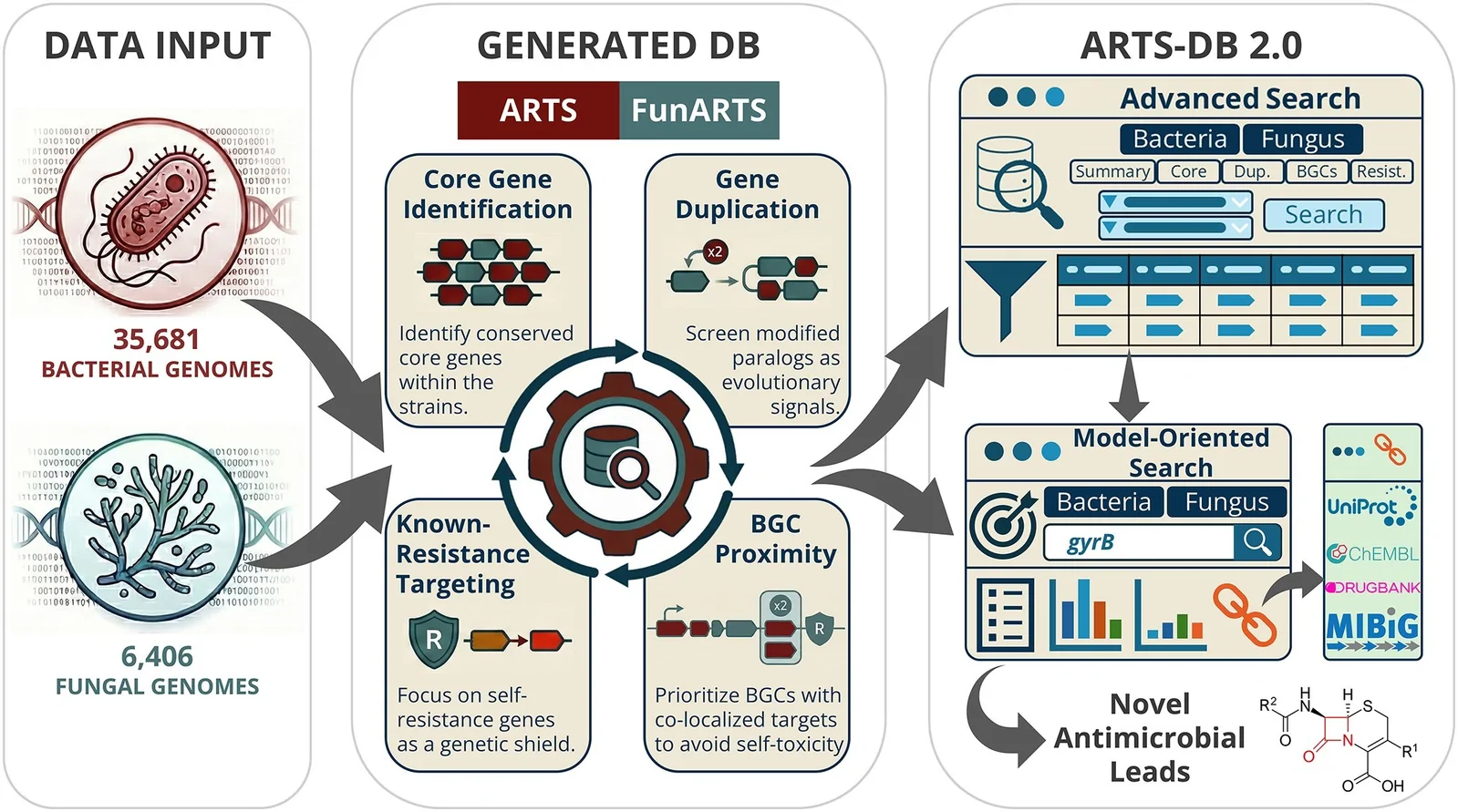

## Introduction

The rapid emergence of antimicrobial resistance (AMR) constitutes one of the most pressing global health challenges of the 21st century, necessitating the urgent discovery of novel therapeutic agents [1]. Microbial secondary metabolites have historically served as the most prolific source of antibiotics and bioactive compounds. However, traditional screening methods based on bioactivity have increasingly suffered from high rates of rediscovery, prompting a shift towards genomics-driven approaches, commonly referred to as genome mining [2]. This approach focuses on the computational analysis of genome sequences to identify biosynthetic gene clusters (BGCs), which are co-localized sets of genes encoding the enzymes required to produce specialized metabolites (e.g. antibiotics). With millions of predicted BGCs now detectable across genomic and metagenomic datasets [3], genome mining is indispensable for triaging clusters and focusing experimental work on the most promising candidates.

Target-directed genome mining (TDGM) prioritizes BGCs by searching for self-resistance genes located within or adjacent to the cluster [4]. In some cases, this resistance is a second, resistant copy of the cellular target that the cluster’s encoded compound inhibits. The duplicated target gene is often sequence-diverged so the compound binds less efficiently, while the protein remains functional. This enables bioactivity prediction even when the compound structure is unknown. Accordingly, a BGC that encodes a duplicated, resistant copy of an essential gene is a strong indicator that the cluster product inhibits that specific target. Classic examples include the acyldepsipeptide (ADEP) locus, where ADEP inhibits ClpP and a cluster-associated accessory *clpP* copy is linked to self-resistance [5], and the salinosporamide A locus, where salinosporamide is a proteasome inhibitor and a redundant proteasome β-subunit (*salI*) encoded in the BGC confers resistance by providing a less susceptible target variant [6]. Conversely, TDGM can be applied in a target-first manner to identify BGCs likely to encode antibiotics acting on a desired target, and to prioritize candidates with novel target variants that may indicate new chemotypes or binding modes.

Antibiotic Resistant Target Seeker (ARTS) automates TDGM by detecting BGC-linked duplicated housekeeping or essential genes and by leveraging the unusual phylogenetic signal that such duplicated targets often show as an indicator of selection associated with self-resistance [7–9], and FunARTS extends TDGM to fungal genomes [10,11]. However, applying these analyses comparatively across thousands of genomes remains computationally intensive and technically demanding, which has limited broad, cross-organism exploration of TDGM signals. To address this bottleneck for bacteria, we previously introduced ARTS-DB [12] as a repository of precomputed ARTS results. In contrast, while FunARTS enables TDGM on individual fungal genomes, a comparable resource providing precomputed, queryable TDGM outputs at scale for fungi has been lacking.

Here, we present ARTS-DB 2.0, a substantially expanded and upgraded release that unifies bacterial and fungal TDGM outputs in a single platform and enables interactive exploration without rerunning genome-mining pipelines. ARTS-DB 2.0 now covers 42 087 non-redundant genomes (35 681 bacterial genomes and 6406 curated fungal assemblies) and incorporates updated biosynthetic annotations with antiSMASH 8.0 [13], alongside an expanded set of resistance models including the NCBI-AMR HMM Catalog [14]. We additionally expand Model-Oriented Search module for gene model-based exploration and provide extensive cross-links to UniProt [15], MIBiG [16], and DrugBank [17]. Together, these updates make ARTS-DB 2.0 a precomputed resource for resistance-aware genome mining in bacteria and fungi, accessible through a web interface for query, filtering, and comparative analysis.

## Materials and methods

### Selection of bacterial and fungal genomes

The ARTS-DB 2.0 was expanded to encompass both bacterial and fungal domains using distinct data acquisition strategies to ensure high-quality input for genome mining.

To maintain high assembly quality and interoperability with established repositories, bacterial genomes were sourced from the antiSMASH-DB v4 [18]. This repository was specifically chosen over raw public databases because its rigorous internal filtration steps ensure a highly curated and selective dataset. A total of 35 726 bacterial genomes were initially retrieved. These genomes were subsequently mapped to the ARTS internal reference sets for phylogenetic grouping through a semi-manual curation process. During this process, 45 genomes that could not be assigned to a valid reference group were excluded. The final bacterial dataset consists of 35 681 genomes.

Fungal genomes were retrieved from the NCBI GenBank Release 267 [19] (accessed on 15 June 2025). We employed a two-tiered selection strategy to balance taxonomic breadth with data quality.

First, to ensure comprehensive coverage of fungal diversity, we included all genomes designated as ‘Reference Genomes’ by NCBI [20]. This step yielded 5107 genomes representing the core taxonomic diversity.

Second, for the remaining non-reference genomes, we applied a strict filtering and dereplication pipeline aligned with the curation standards of antiSMASH-DB v4 to avoid saturating the database with redundant results from nearly identical strains (e.g. those differing by only single nucleotide polymorphisms). The filtering process proceeded as follows:

*Assembly Quality Filter:* We initially selected genomes with an assembly level of ‘Complete genome’, ‘Chromosome’, and ‘Scaffold’ resulting in a pool of 10 457 genomes.

*Fragmentation Filter:* To avoid the impact of highly fragmented assemblies on BGC prediction quality, we retained only genomes with fewer than 150 contigs. This step reduced the pool to 3232 genomes.

*Dereplication:* We calculated all-vs.-all genomic distances using MASH [21] to identify redundant entries. Using a distance cutoff of 0.004 (corresponding approximately to ≥99.6% average nucleotide identity), we clustered similar genomes and selected a single representative for each cluster based on the following priority hierarchy: (i) lowest contig count, (ii) highest assembly level (Complete genome > Chromosome > Scaffold), and (iii) the most recent submission date. This dereplication step resulted in 1299 representative genomes.

Taken together, the curated dataset for ARTS-DB 2.0 comprises a total of 42 087 non-redundant genomes from biological isolates. This collection includes the 35 681 bacterial genomes sourced from antiSMASH-DB and the 6406 fungal genomes processed through the redundancy filtering pipeline. By integrating these high-quality assemblies, the updated database significantly expands its taxonomic scope, providing a robust foundation for comparative genome mining across both bacterial and fungal lineages.

### Fungal gene annotation

While the previous version of antiSMASH 7.0 included internal fungal gene finding via GlimmerHMM [22], the updated antiSMASH 8.0 workflow requires pre-annotated input for fungal sequences [13]. Therefore, to ensure compatibility with the version 8 pipeline, we performed *de novo* gene annotation for the 4259 fungal genomes in our dataset that lacked suitable annotations in their GenBank records.

We utilized the Funannotate2 pipeline [23] for genome preparation, gene prediction, and functional annotation. The structural gene prediction was conducted using a consensus approach that integrated multiple *ab initio* algorithms, including AUGUSTUS [24], GeneMark-ES/ET [25,26], SNAP [27], GlimmerHMM [22], and tRNAscan-SE [28]. To further enhance prediction accuracy, we generated deep learning-based evidence by running Helixer [29] independently of the Funannotate2 pipeline. The resulting gene models from Helixer (in GFF3 format) were then incorporated into the Funannotate2 as high-weight evidence during the prediction stage.

Following structural gene prediction, the functional annotation module within Funannotate2 was utilized to assign biological context to the generated gene models. This automated workflow also integrates searches against several key databases, including Pfam [30], UniProtKB/Swiss-Prot [15], and BUSCO [31], to comprehensively characterize the fungal proteomes. This workflow produced fully annotated GenBank files for 4259 fungal genomes, which were subsequently prepared for antiSMASH 8.0 [13] and FunARTS [11] analysis.

### ARTS and FunARTS analysis

We employed modified versions of the ARTS [9] and FunARTS [11] pipelines, in which incorporated tools such as antiSMASH were updated to more recent versions, to execute TDGM on the respective datasets. The primary objective of these tools is to identify essential core genes, detect duplication events, and facilitate the prioritization of BGCs based on their genomic proximity to these targets.

The bacterial dataset was processed using the ARTS pipeline to screen for core genes, gene duplication (Core genes over pre-computed thresholds), BGC proximity (Core genes intersecting with BGC boundaries), phylogeny [Core genes that show evidence of horizontal gene transfer (HGT)], and known resistance factors. To enhance core TDGM metrics with evolutionary context and specific resistance markers, we activated the phylogeny generation module (utilizing ASTRAL [32]) and the known resistance screening module by employing the arguments *‘—astral—options phyl, kres*.’

We used FunARTS to analyse the fungal dataset. This workflow was very similar to the criteria used for bacterial analysis, except for phylogenetic screening. Therefore, the pipeline was run with the *‘—options kres*’ argument to turn on the known resistance screening module and leave out the phylogenetic steps.

As the initial step for both pipelines, BGCs were identified using antiSMASH 8.0 [13]. This was triggered directly within the ARTS and FunARTS workflows via the ‘*—runantismash*’ argument. To ensure high-resolution screening and extensive comparative analysis, antiSMASH was executed with the following parameters: *‘- -cb-knownclusters - -cb-subclusters—clusterhmmer - -cc-mibig—cb-general*.’

For resistance gene screening, we expanded the detection capabilities in this version. In addition to the internal ‘known resistance’ hidden Markov models (HMMs) inherent to the ARTS/FunARTS frameworks (passed via ‘*—knownhmms*’), we integrated the NCBI AMR Reference HMMs used by AMRFinderPlus [14] as custom models. These external models were incorporated into the analysis using the ‘*—customhmms*’ argument, allowing for a broader identification of putative resistance factors across both lineages.

### External database mapping

To enhance the interoperability of ARTS-DB 2.0 and provide extensive biological and pharmacological context, we established cross-links with seven external repositories. These mappings were conducted at two distinct levels: BGCs and individual target gene models.

Predicted BGCs were cross-referenced with global cluster families to provide evolutionary context. We mapped the BGC regions identified by antiSMASH to the BiG-FAM database [33] using BiG-SLiCE 1.0.0 [34], and to PanBGC-DB [35] by identifying regions with 100% sequence identity via exact hash matching. Through this screening, we identified the specific gene cluster families (GCFs) associated with each predicted cluster, allowing users to explore relationships between the detected BGCs and wider biosynthetic families.

We also implemented a comprehensive mapping strategy for the underlying gene models used in the ARTS and FunARTS. The models were first mapped to the UniProt database by cross-referencing model IDs [15] to retrieve detailed protein structure and functional information. Using the UniProt identifiers as a bridge, we extracted associated pharmacological data from ChEMBL [36], DrugBank [17], and DrugCentral [37]. Additionally, links to known validated clusters in the MIBiG database [16] were established through sequence similarity searches, retaining the best-matching sequences for each gene model.

This integration enables users to seamlessly navigate from a predicted resistance gene in ARTS-DB 2.0 to its corresponding protein structure, potential drug ligands, and known chemical interactions in external databases.

### Database construction and web server

ARTS-DB 2.0 is implemented using the Flask web framework (https://flask.palletsprojects.com/), combining a lightweight SQLite database with JSON-based auxiliary files to organize precomputed ARTS and FunARTS results. SQLite (https://www.sqlite.org/) stores curated data for fast retrieval, including core gene hits, BGC proximity records, and summary statistics, thereby powering the interactive Query Builder interface. Detailed per-genome ARTS/FunARTS outputs are retrieved directly from precomputed result directories on the server, enabling fast access without on-the-fly computation. Cross-links to external repositories are maintained as compact JSON mappings, which also support the Model-Oriented Search module. The platform is deployed using Gunicorn (https://gunicorn.org/) as the WSGI server behind an Nginx reverse proxy (https://nginx.org/), ensuring stable performance, secure request handling, and reliable delivery of static result files.

## Results and discussion

### Database content and phylogenetic distribution

**Figure 1:**
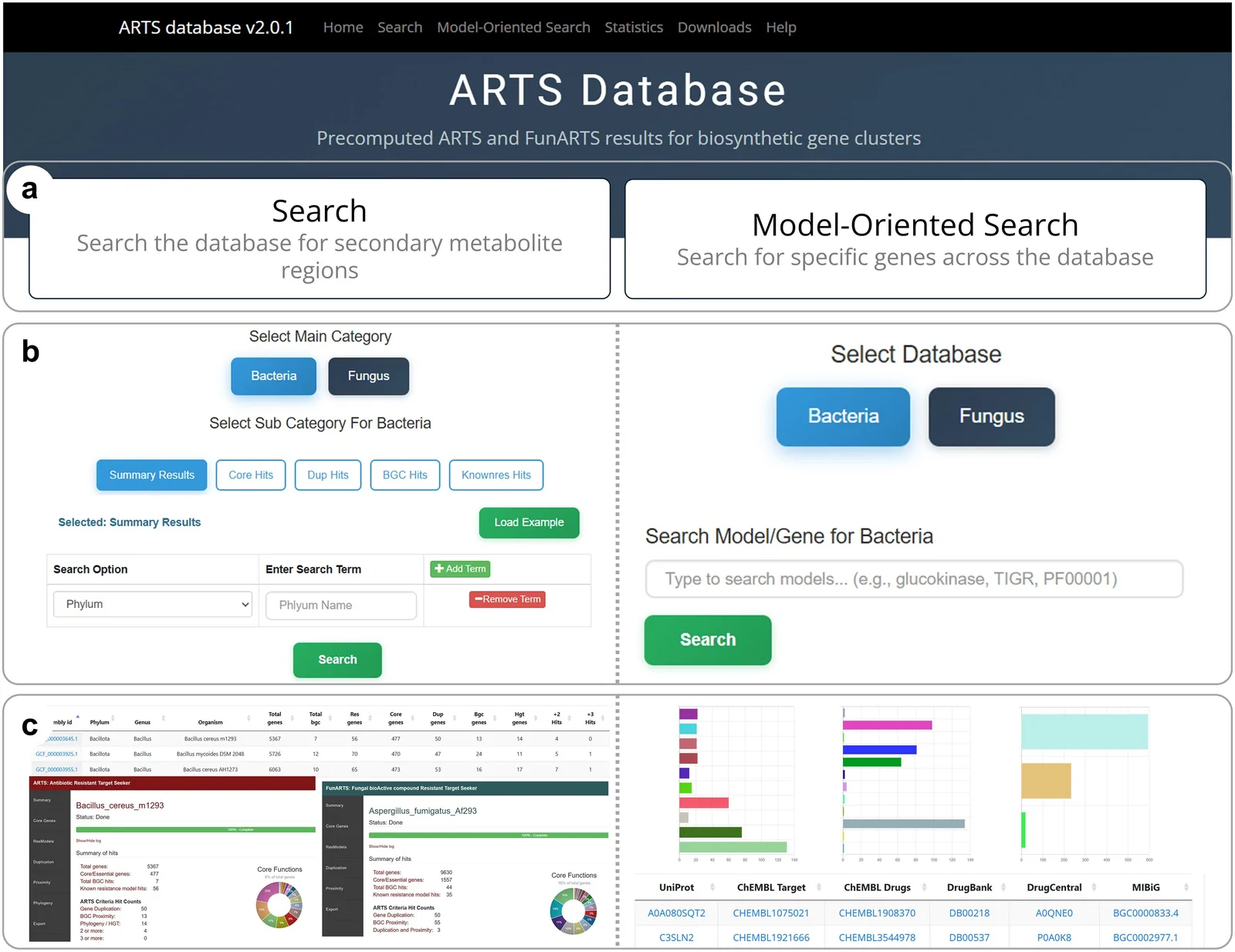
Overview of the ARTS-DB 2.0 web interface and search workflows. (a) The landing page provides two main entry points: the ‘Search’ module for exploring precomputed ARTS and FunARTS results using user-defined screening criteria, and the ‘Model-Oriented Search’ for gene-centred exploration across the database. (b) Query interfaces for the two search modes. The Search module allows users to select the bacterial or fungal dataset and combine multiple filtering criteria, whereas the Model-Oriented Search enables direct querying of specific gene models. (c) Representative output pages. Search results are presented as interactive Tables with links to individual ARTS or FunARTS genome reports, while Model-Oriented Search results summarize gene-associated distributions and provide cross-links to external biological and pharmacological resources.

ARTS-DB was originally developed to make bacterial TDGM results searchable without rerunning ARTS on individual genomes. However, fungal TDGM outputs were not available as a comparable precomputed resource, and the rapid growth of genomic data created a strong need for expanded coverage and updated biosynthetic annotation. We therefore extended the resource to ARTS-DB 2.0, which integrates precomputed ARTS (bacterial) and FunARTS (fungal) outputs and substantially increases genome coverage. In addition, ARTS-DB 2.0 incorporates updated BGC annotations generated with antiSMASH 8.0, expands resistance model integration, and improves usability through a redesigned web interface, bug fixes, and direct, transparent navigation from summary views to individual ARTS/FunARTS results (Fig. 1).

**Figure 2:**
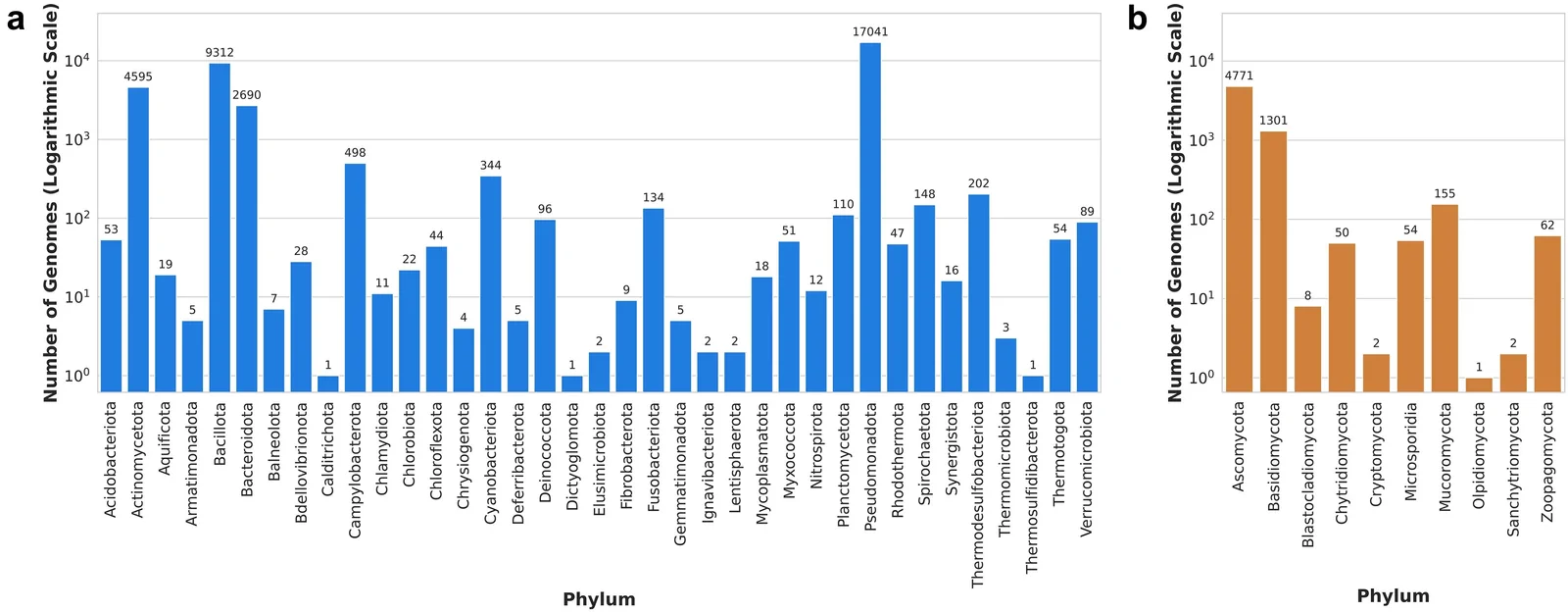
Taxonomic distribution of bacterial and fungal genomes by phylum. Bar charts show the number of genomes assigned to each phylum on a logarithmic scale. (a) Distribution of the 35 681 bacterial genomes, with Pseudomonadota, Bacillota, and Actinomycetota representing the most abundant phyla. (b) Distribution of the 6406 fungal genomes, dominated by Ascomycota and Basidiomycota.

The curated dataset currently encompasses a total of 42 087 genomes, consisting of 35 681 bacterial and 6406 fungal assemblies. The bacterial subset provides a robust representation of the bacterial domain, with the phyla Pseudomonadota, Bacillota, and Actinomycetota constituting the majority of entries (Fig. 2a, Supplementary Table S1). The fungal dataset is dominated by Ascomycota and Basidiomycota, ensuring that the most biosynthetically active fungal lineages are well-represented (Fig. 2b, Supplementary Table S4).

The ARTS and FunARTS analyses revealed distinct distribution patterns of TDGM hits across the two lineages (Table 1, Supplementary Tables S2 and S3, Supplementary Tables S5 and S6). Reflecting their larger genome size and eukaryotic complexity, fungal genomes exhibited significantly higher counts in fundamental genomic metrics. The average number of identified core genes per fungal genome was 1 470.65, ~3.3 times higher than the bacterial average of 442.42. Similarly, duplication events and BGC-proximal genes were far more prevalent in fungi, with averages of 85.3 and 47.5 per genome, respectively, compared to 25.04 and 19.30 in bacteria.

**Table 1:** Comparative overview of genomic datasets and TDGM metrics in ARTS-DB 2.0.

	BACTERIA (*n* = 35 681)		FUNGI (*n* = 6406)	
Metric/category	Total counts	Mean/genome	Total counts	Mean/genome
**Identified core genes**	15 785 898	442.42	9 421 008	1470.65
**Duplicated core genes**	893 457	25.04	546 455	85.30
**BGC proximity**	688 500	19.30	304 309	47.50
**Known resistance hits**	1 335 931	37.44	206 668	32.26
**Phylogeny hits**	2 600 749	72.89	N/A*	N/A*
**Criteria hits ≥ 2**	496 272	13.91	36 540	5.70
**Criteria hits ≥ 3**	32 188	0.90	N/A*	N/A*

The Table summarizes the total counts and per-genome averages for key mining criteria across bacterial and fungal datasets. ‘*n*’ indicates the total number of genomes in the database. ‘Mean/Genome’ represents the average number of hits identified per individual genome. Criteria Hits ≥ 2 and Criteria Hits ≥ 3 indicate targets satisfying multiple ARTS or FunARTS criteria simultaneously (e.g. Duplication, BGC Proximity, or Phylogeny Hits). *Phylogenetic screening (and thus HGT detection) was not applied, as it is currently not supported by the FunARTS workflow.

In contrast, resistance profiling revealed a different pattern. Despite lower per-genome core gene counts, bacteria displayed a slightly higher density of known resistance hits (37.44 hits/genome) compared to fungi (32.26 hits/genome), likely reflecting the bacterial bias inherent in current resistance gene models. Additionally, while fungi are rich in potential core gene targets, the co-localization of known resistance genes with BGCs is less frequent. This highlights both the selectivity of the pipeline and the need to expand fungal-specific resistance models.

### Expanded functionality and interoperability

A fundamental enhancement in ARTS-DB 2.0 is the migration of the biosynthetic detection engine from antiSMASH v5 to the state-of-the-art version 8 [13]. This upgrade significantly elevates the resolution and scope of BGC prediction, increasing the diversity of detectable cluster classes from ~60 distinct types in the previous version to 101 types in the current release. Consequently, this expansion captures chemical classes, such as benzoxazoles, deazapurines, and fungal NRPS-like lysine biosynthesis, while simultaneously refining the detection of established categories such as NRPS and PKS. These improvements ensure that ARTS-DB 2.0 provides a far more detailed and comprehensive map of the secondary metabolite landscape than was previously possible.

In addition to improved biosynthetic profiling, we have significantly broadened the range of resistance gene screening. We integrated 772 additional models from the NCBI-AMR HMM Catalog [14] into the ARTS and FunARTS processes. As a result of this integration, the database now provides a more various resolution for known resistance mechanisms, such as vancomycin resistance clusters and 16S rRNA methyltransferases, while also identifying new targets, such as bleomycin binding proteins and fosfomycin resistance glutathione transferases.

Finally, ARTS-DB 2.0 has greatly enlarged its cross-links to external repositories to enhance data interoperability and facilitate drug discovery. At the cluster level, predicted BGCs are mapped to BiG-FAM and PanBGC-DB to provide evolutionary context within GCFs. For gene-specific insights, models are cross-referenced with MIBiG, thereby linking targets to well-known BGCs and their known natural products. Moreover, direct mapping to UniProt, ChEMBL, DrugBank, and DrugCentral connects specific gene models to protein structures and pharmacological data, allowing researchers to rapidly explore known ligands and their mechanisms of action. Quantifying this, 1453 bacterial models feature external cross-links (1016 mapped to protein structures, 1327 to known ligands, and 890 to both). In fungi, 174 models have cross-links (169 to ligands, but only 13 to structures). This lower structural coverage in fungi reflects the historical bias of public databases towards bacterial targets, a limitation that will automatically resolve as external fungal structural data expands.

To facilitate reproducibility and support downstream analysis, ARTS-DB 2.0 ensures full data accessibility. All generated datasets—including results Tables, detailed ARTS/FunARTS output files, and external database mapping records—are readily available for download. This feature allows researchers to easily retrieve pre-computed results and integrate them into their custom workflows for further investigation.

### User interface and case studies

The ARTS-DB 2.0 web interface is designed to facilitate rapid search through two distinct exploration modules: the Advanced Search and the Model-Oriented Search.

The Advanced Search enables users to construct complex, multi-layered queries across the entire database. This module retains the four primary screening categories from the previous version: (i) Summary Results (genome-wide statistics), (ii) Core Hits (essential genes), (iii) Duplication Hits (copy number variation), and (iv) BGC Hits (cluster proximity). Additionally, this version introduces a dedicated ‘Known Resistance Hits’ category. This new module allows users to specifically screen for resistance factors. Moreover, users can utilize detailed filters to obtain context-specific data, such as the presence of a resistance gene within a BGC. Additionally, the Tables generated via the ‘BGC Hits’ category contain cross-references with BiG-FAM and PanBGC-DB, thus enabling users to directly access the evolutionary relationships of priority clusters from the results Table.

The search logic is highly flexible; queries can be refined using the ‘Add Term’ function to stack specific parameters, such as taxonomic constraints (Phylum/Genus), gene descriptions, and fundamental ARTS/FunARTS criteria (e.g. duplication status, BGC types, and presence of HGT evidence). Users can further refine these searches by targeting specific HMM-based profiles used for core gene or known resistance gene detection (e.g. TIGRFAM/BUSCO models for core genes) and by filtering genes based on their hit types. These categories include: (i) **Core** (a gene identified as a fundamental core component), (ii) **CoreRes** (a known resistance model hit mapping to the same locus as a core gene within a BGC), (iii) **ResModel** (a known resistance model hit identified and localized within a BGC), and (iv) **ResGene** (a standalone known resistance model hit located outside of a BGC). Collectively, they allow users to distinguish between resistance mechanisms embedded within biosynthetic contexts and those occurring as standalone genomic features. These parameters can be applied individually to both bacterial and fungal datasets. Results are presented in dynamic Tables that allow for sorting, searching, and direct exporting of data for downstream analysis.

Complementing the structured filtering of the Advanced Search, the Model-Oriented Search offers a gene-centric perspective. By inputting a specific gene of interest (e.g. *rpoB, gyrB* or *dnaN*), users can instantly visualize its global distribution across the database. The results dashboard provides interactive charts summarizing the gene’s proximity to various BGC types, its duplication frequency across different phyla, and its alignment with TDGM criteria. Furthermore, the interface features a dedicated ‘External Resources’ section, where specific gene models are directly linked to MIBiG, UniProt, ChEMBL, DrugBank, and DrugCentral. This integration allows users to seamlessly transition from genomic hit distribution to detailed structural and pharmacological data. This mode is particularly valuable for researchers starting with a specific target hypothesis rather than a specific organism.

The following case studies illustrate typical usage scenarios of ARTS-DB 2.0 and highlight how different query strategies can be applied to prioritize resistance-associated biosynthetic targets in bacteria and fungi.

**Figure 3:**
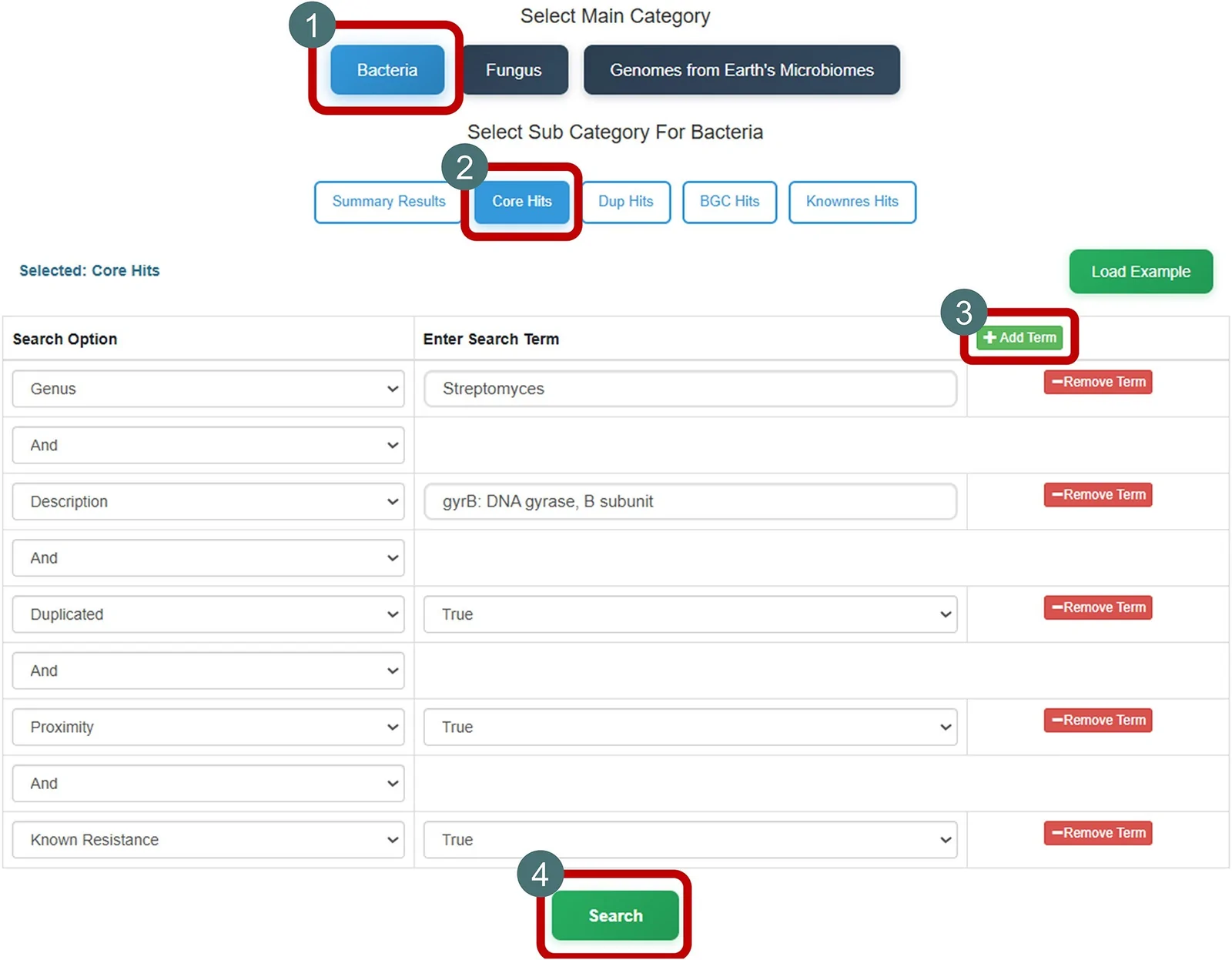
Step-by-step workflow of the Search module. The interface allows users to construct complex, multi-layered queries. The numbered steps highlight the logical flow: (1) the user selects the main category. (2) A suitable sub-category is selected based on the intended screening strategy. (3) To deepen the analysis, the ‘Add Term’ button is used to incorporate additional search options. (4) The Search button executes the query, retrieving results that match all defined criteria.

*Case Study 1–Searching Core Hits for Specific Resistance Candidate:* To demonstrate the bacterial target prioritization workflow, the DNA gyrase subunit B (*gyrB*) within the genus *Streptomyces* serves as a representative case study (Fig. 3). Since GyrB is a validated target for aminocoumarin antibiotics, identifying resistant variants can lead to the discovery of novel producers [38]. For users aiming to assess the broad genomic landscape, the Summary Results option can be utilized; filtering for the genus ‘*Streptomyces*’ instantly yields statistical summaries for all 779 registered strains. For the targeted identification of specific genomes containing the *gyrB* core gene, the Core Hits subcategory is applied. Here, a multi-parametric query is constructed using the ‘Add Term’ function: restricting the search to *Streptomyces*, specifying *gyrB* as the target description, and desiring the simultaneous presence of three key resistance signals (Duplicated, Proximity, and Known Resistance all set to ‘True’). Remarkably, this stringent query reduces the broad dataset to 8 specific *Streptomyces* strains in seconds. This result highlights the platform’s efficiency in filtering thousands of genomes to identify a precise subset of organisms where *gyrB* exhibits strong, multi-layered evidence of resistance potential.

*Case Study 2–Fungal Resistance Exploration:* To illustrate the application of ARTS-DB 2.0 in fungal target mining, the proteasome subunit within the fungal genus *Aspergillus* serves as a case study. For this analysis, the KnownRes Hits subcategory is utilized to specifically query resistance determinants based on their genomic context. Furthermore, the search can be refined based on hit types. To identify *Aspergillus* genomes where proteasome subunits are co-localized with BGCs, a structured query is constructed. The parameters are defined by setting the Genus to ‘*Aspergillus*,’ the Gene Description to ‘proteasome,’ and the Hit Type to ‘CoreRes.’ This targeted search effectively filters the fungal dataset, retrieving 104 *Aspergillus* entries that satisfy the strict co-localization criteria, thereby demonstrating the platform’s capacity to precisely identify putative resistance targets.

**Figure 4:**
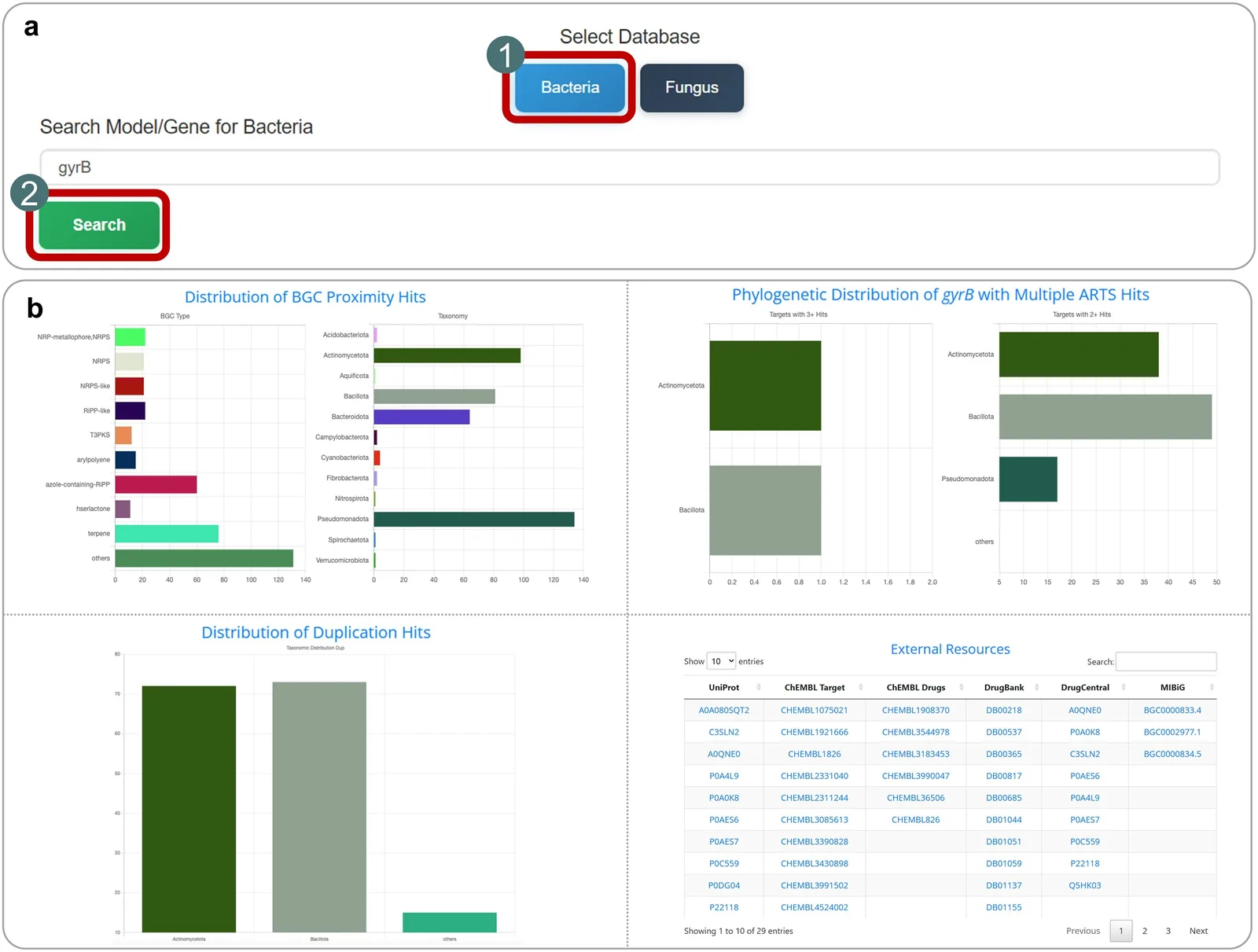
Workflow and output of the Model-Oriented Search module. (a) (1) Designed for rapid, model-based exploration, the user first selects the target taxonomic domain (Bacteria or Fungus) to define the scope of the dataset. (2) A specific gene name (e.g. *gyrB*) is then entered, and the search is executed. (b) The resulting gene-specific dashboard displays statistical distributions for BGC proximity, duplication, and multiple ARTS hits across taxa, alongside a Table with cross-links to external resources.

*Case Study 3–Model Oriented Search for Specific Gene Model:* To demonstrate the gene-centric exploration capabilities of ARTS-DB 2.0, we applied the Model-Oriented Search module to the DNA gyrase subunit B gene (*gyrB*), a well-established antibacterial target (Fig. 4). While the previous case studies focus on organism- or resistance-centric queries, the Model-Oriented Search enables users to explore a specific gene model across the entire database. The analysis begins with the selection of the taxonomic domain, followed by querying the gene name of interest. In this example, the bacterial dataset was selected, and *gyrB* was queried. The resulting overview provides summary information on the selected gene model, along with visualizations describing its phylogenetic distribution, duplication frequency, association with BGC types, and conformity with TDGM criteria. As highlighted by these visualizations, *gyrB* is predominantly distributed within the phyla Actinomycetota, Bacillota, and Pseudomonadota, with the charts exhibiting resistance-associated signals. In addition, the Model-Oriented Search integrates direct links to external resources, including UniProt, MIBiG, DrugBank, ChEMBL, and DrugCentral, enabling rapid access to functional, biosynthetic, and pharmacological information. Together, this case study highlights how ARTS-DB 2.0 supports rapid, large-scale evaluation of predefined target genes within a resistance-aware genome mining framework.

### Validation of known targets

To evaluate whether ARTS-DB 2.0 recovers established resistance-associated biosynthetic systems, we queried the database for validated bacterial and fungal targets. A search for duplicated, BGC-associated *lspA* identified 64 bacterial entries, including the myxovirescin producer *Myxococcus xanthus DK1622* [39]. Similarly, a fungal search for duplicated, BGC-associated ribosomal protein L10 retrieved 28 entries, including the ligustrone A-producing lineages *Penicillium paradoxum* and *Botryosphaeria dothidea* [40]. These examples demonstrate that ARTS-DB 2.0 can recover experimentally validated target–BGC associations in both bacteria and fungi.

The platform can also support target-centred exploration across lineages. Searches for proteasome subunits retrieved bacterial and fungal producers of known proteasome inhibitors, including *Salinispora tropica*, which produces salinosporamide A [6], and *Aspergillus nidulans*, which produces fellutamide B [41]. Associated MIBiG entries and external database links can be accessed through the Model-Oriented Search, connecting predicted targets with known natural products and BGCs.

### Limitations

ARTS-DB 2.0 provides precomputed results generated by the ARTS and FunARTS pipelines and therefore shares the methodological limitations described for these tools. TDGM is based primarily on resistance-associated signals such as the duplication of essential genes and their proximity to BGCs. However, only a subset of BGCs encode an identifiable resistant copy of the cellular target. Other self-resistance mechanisms, including efflux, compound modification, target protection, or resistance-conferring point mutations, may not be detected and can therefore lead to false-negative results.

Conversely, the presence of a duplicated core gene close to a predicted BGC does not by itself establish a role in self-resistance. Gene duplication may have other biological functions, and some genes located within or near a BGC may participate in biosynthesis rather than resistance, potentially resulting in false-positive predictions. Interpretation is also affected by uncertainty in predicted BGC boundaries, particularly when relevant genes lie close to the edges of an antiSMASH-defined region.

Additional limitations arise from the currently available reference models and the taxonomic composition. Known-resistance models remain biased towards well-characterized bacterial systems, while fungal resistance determinants are less comprehensively represented. In addition, the bacterial and fungal datasets are dominated by a few extensively sequenced phyla. Approximately 94% of bacterial genomes belong to four phyla, while Ascomycota and Basidiomycota comprise ∼95% of fungal genomes. As a result, the statistical summaries, particularly in the Model-Oriented Search, reflect this sampling bias and may not represent resistance target distributions across underrepresented lineages.

ARTS-DB results should therefore be regarded as prioritization hypotheses rather than definitive assignments of resistance function or compound target. As reference datasets, genome annotations, BGC predictions, and the underlying ARTS and FunARTS methods improve, these advances will be incorporated into future versions of the database.

## Conclusion

ARTS-DB 2.0 substantially extends the original platform by unifying large-scale bacterial and fungal genome mining within a single target-directed framework. With the integration of more than 42 000 high-quality genomes and the inclusion of FunARTS, the database enables systematic exploration of self-resistance-associated biosynthetic signals across two major biological lineages. The upgrade to antiSMASH 8.0 improves the diversity of BGC predictions, while the incorporation of NCBI AMR HMMs expands resistance gene detection.

In addition to analytical advances, ARTS-DB 2.0 strengthens data interoperability and usability through extensive cross-links to major biological and pharmacological databases and through flexible query and gene-centric exploration interfaces. Overall, ARTS-DB 2.0 provides a scalable and integrative resource for prioritizing BGCs and resistance-associated targets in bacteria and fungi, supporting future natural product discovery and mode-of-action studies.

## Supplementary Material

baag060_Supplemental_File

## Data Availability

The ARTS-DB web interface is freely accessible to all users without any login or registration requirements at https://arts-db.cs.uni-tuebingen.de. All underlying data supporting the findings of this study, including the precomputed results and datasets used for the case studies, are publicly available and can be downloaded directly from https://arts-db.cs.uni-tuebingen.de/downloads.
