# Supplementary material for: ARTS-DB 2.0: an expanded database for target-directed genome mining across bacteria and fungi
Source: Database (Oxford). 2026 Sep 25;2026:baag060. doi: 10.1093/database/baag060 (PMC13613211; doi:10.1093/database/baag060)
Supplement: Supplementary file 1 [file baag060_supplemental_file.pdf]

SUPPLEMENTARY INFORMATION

ARTS-DB 2.0: An Expanded Database for Target-Directed Genome Mining Across Bacteria and Fungi

Turgut Mesut Yilmaz[1,2], Semih Erdogmus[1,2], Caner Bağcı[1,2], Nadine Ziemert[1,2,*]

[1] Translational Genome Mining for Natural Products, Interfaculty Institute of Microbiology and Infection Medicine Tübingen (IMIT), Interfaculty Institute for Biomedical Informatics (IBMI), University of Tübingen, Tübingen, Baden-Württemberg 72076, Germany
[2] German Center for Infection Research (DZIF), Partner Site Tübingen, 72076, Tübingen, Germany

* To whom correspondence should be addressed. Email: nadine.ziemert@uni-tuebingen.de

Supplementary Table S1: Bacterial entries by phylum

Phylum	Count	Phylum	Count
Acidobacteriota	53	Fibrobacterota	9
Actinomycetota	4595	Fusobacteriota	134
Aquificota	19	Gemmatimonadota	5
Armatimonadota	5	Ignavibacteriota	2
Bacillota	9312	Lentisphaerota	2
Bacteroidota	2690	Mycoplasmatota	18
Balneolota	7	Myxococcota	51
Bdellovibrionota	28	Nitrospirota	12
Calditrichota	1	Planctomycetota	110
Campylobacterota	498	Pseudomonadota	17041
Chlamydiota	11	Rhodothermota	47
Chlorobiota	22	Spirochaetota	148
Chloroflexota	44	Synergistota	16
Chrysiogenota	4	Thermodesulfobacteriota	202
Cyanobacteriota	344	Thermomicrobiota	3
Deferribacterota	5	Thermosulfidibacterota	1
Deinococcota	96	Thermotogota	54
Dityoglomota	1	Verrucomicrobiota	89
Elusimicrobiota	2		

Supplementary Table S2: Bacterial mining metrics and top performers

	Total Counts	Mean / Genome	Genome with Highest Count
**Identified Core Genes**	15,785,898	442.42	Escherichia coli O55:H7 GCF_013167935.1 – 644
**Duplicated Core Genes**	893,457	25.04	Pseudoalteromonas sp. 2CM39R GCF_023161845.1 – 500
**BGC Proximity**	688,500	19.30	Xenorhabdus_budapestensis GCF_017743015.1 – 107
**Known Resistance Hits**	1,335,931	37.44	Providencia stuartii GCF_023547145.1 – 164
**Phylogeny Hits**	2,600,749	72.89	Roseibacterium_beibuensis GCF_025000125.1 – 570
**Criteria Hits ≥ 2**	496,272	13.91	Roseibacterium_beibuensis GCF_025000125.1 – 336
**Criteria Hits ≥ 3**	32,188	0.90	Roseibacterium_beibuensis GCF_025000125.1 – 36

Supplementary Table S3: Distribution of BGC Proximity Hits in Bacterial genomes by BGC types

Bgc Types	BGC Proximity Counts	Bgc Types	BGC Proximity Counts
Terpene	249454	cyclic-lactone-autoinducer	6532
NRPS	123995	Resorcinol	6209
RiPP	99876	NRPS/Phenazine/Metallophore	5078
PKS	87566	NRPS/RiPP	4464
Metallophore	47562	Ectoine	3770
NRPS/Metallophore	44437	Arylpolyene/Resorcinol	3535
Arylpolyene	36827	Terpene/Betalactone	3451
Betalactone	27662	NRPS/PKS/Metallophore	3026
Redox Cofactor	25808	Phosphonate	3003
Others	24364	NRPS/Betalactone	2584
Ni Siderophore	20273	PKS/RiPP	2527
NRPS/PKS	20182	Terpene/RiPP	2248
hserlactone	14068	NRPS/Terpene/Metallophore	1689
NAPAA	9540	NRPS/Redox Cofactor	1604
NRPS/Terpene	7390	NRPS/PKS/RiPP	1284
Terpene/PKS	6623	NRPS/PKS/Terpene	1137

Supplementary Table S4: Fungal entries by phylum

Phylum	Count	Phylum	Count
Ascomycota	4771	Microsporidia	54
Basidiomycota	1301	Mucoromycota	155
Blastocladiomycota	8	Olpidiomycota	1
Chytridiomycota	50	Sanchytriomycota	2
Cryptomycota	2	Zoopagomycota	62

Supplementary Table S5: Fungal mining metrics and top performers

	Total Counts	Mean / Genome	Genome with Highest Count
**Identified Core Genes**	9,421,008	1,470.65	Phanerochaete_sordida GCA_019973155.2 – 1,657
**Duplicated Core Genes**	546,455	85.30	Rhodosporidiobolus_fluvialis GCA_041531625.1 – 1,579
**BGC Proximity**	304,309	47.50	Talaromyces_proteolyticus GCA_021365285.1 207
**Known Resistance Hits**	206,668	32.26	Basidiobolus_ranarum GCA_040143965.1 – 91
**Criteria Hits ≥ 2**	36,540	5.70	Ramulariopsis_pseudoglycines GCA_043513825.1 – 134

Supplementary Table S6: Distribution of BGC Proximity Hits in Fungal genomes by BGC types

Bgc Types	BGC Proximity Counts	Bgc Types	BGC Proximity Counts
**Terpene**	124113	RiPP	1457
**NRPS**	109295	NAPAA	789
**PKS**	77533	NRPS/Betalactone	775
**NRPS/PKS**	17497	Phosphonate	462
**Betalactone**	7078	NRPS/RiPP	130
**Metallophore**	6381	NRPS/PKS/Metallophore	123
**NRPS/Metallophore**	6381	PKS/RiPP	93
**NRPS/Terpene**	6375	Terpene/Betalactone	91
**Terpene/PKS**	5200	NRPS/Terpene/Metallophore	67
**Ni Siderophore**	2140	NRPS/PKS/RiPP	46
**NRPS/PKS/Terpene**	1742	Terpene/RiPP	36
